# Evaluation of an iELISA for detection and quantification of rabies antibodies in domestic dog sera

**DOI:** 10.1016/j.vaccine.2023.09.004

**Published:** 2023-10-20

**Authors:** Ahmed Lugelo, Katie Hampson, Lorraine M. McElhinney, Felix Lankester

**Affiliations:** a Global Animal Health Tanzania, Arusha, Tanzania; b Boyd Orr Centre for Population and Ecosystem Health, School of Biodiversity, One Health and Veterinary Medicine, University of Glasgow, Glasgow G12 8QQ, UK; c Viral Zoonoses Group, Animal and Plant Health Agency (APHA), Weybridge, New Haw, Addlestone, Surrey KT15 3NB, UK; d Paul G. Allen School for Global Animal Health, Washington State University, Pullman, WA 99164, USA

**Keywords:** Platelia^TM^ Rabies II, Canine rabies, iELISA, FAVN, Rabies serology, Mass dog vaccination

## Abstract

Many rabies endemic-countries have recognized rabies as a public health problem that can be eliminated. As a result, some countries have started implementing small-scale vaccination programs with the aim of scaling them up. Post-vaccination serological monitoring is crucial to assess the efficacy of these programs. The recommended serological tests, the rapid fluorescent focus inhibition test, and the fluorescent antibody virus neutralization (FAVN) are accurate; however, the procedures require considerable expertise and must be carried out in high containment facilities, which are often not available in rabies endemic countries. Given these constraints, enzyme linked immunosorbent assays (ELISAs) have been considered as alternative methods to neutralization tests. This is the first study to evaluate, under field conditions, the performance of the commercial rabies indirect-ELISA (iELISA), the Platelia^TM^ Rabies II kit *ad usum Veterinarium* kit, using sera from domestic dogs. Serum samples were collected from two groups of community dogs in northern Tanzania: i) dogs with no history of vaccination against rabies (n = 100) and ii) dogs vaccinated with the Nobivac Canine Rabies® vaccine (n = 101) four weeks previously. When compared to the gold standard FAVN test, the iELISA was found to be 99% specific and 98% sensitive and there was a significant correlation between the two tests (p < 0.001, r = 0.92). Given these findings, we conclude that the Platelia^TM^ Rabies II kit *ad usum Veterinarium* can be considered a valuable tool for the rapid assessment of vaccination status of animals in vaccination programs.

## 1 Introduction

Vaccination of domestic carnivores is the most effective way to prevent, control and eliminate rabies in areas where domestic dogs are the main source of human infection. Epidemiological and modeling studies have shown that sustaining a coverage of greater than 40% for several years can interrupt transmission of rabies, eventually leading to its elimination [1], [2], [3]. Post-vaccination serological testing provides a means to assess the efficacy of vaccination campaigns, as the presence of virus neutralizing antibodies (VNAs) in serum samples is a reliable indication of successful vaccination [4]. The WHO Expert Committee on rabies has recommended a titre of 0.5 IU/ml as a minimum level of protective VNAs in humans [5], and the same level is used for animals [6].

Currently, the Fluorescent Antibody Virus Neutralization (FAVN) and Rapid Fluorescent Focus Inhibition Test (RFFIT) are the techniques recommended for serum neutralization assays. These methods have high sensitivity and specificity and provide accurate results, but are time consuming and labour intensive [7]. Furthermore, performing these tests requires skilled technicians and the procedure must be done in high containment laboratories, thus limiting their widespread availability [8]. These constraints have prompted the development of enzyme linked immunosorbent assays (ELISAs) as an alternative method to serum neutralization tests. ELISAs have several advantages in that they are relatively quick, can be automated and do not require handling of live virus, thus the procedure can be performed in low-level containment laboratories [9], [10].

Various ELISA kits have been developed to detect rabies antibodies in humans [11], [12], for follow-up investigation in wildlife species, particularly foxes [7], [13] and for domestic carnivores [14], [15]. Though there are many commercialized iELISA kits for rabies serology, only a few of them have been validated and approved for such purpose. An indirect iELISA (SerELISA^TM^ Rabies Ab mono Indirect, Synbiotics) developed by Synbiotics Europe in collaboration with ANSES and Animal and Plant Health Agency became the first iELISA kit to be described by the World Organisation for Animal Health (WOAH) as a rabies serological test for pets involved in international trade [16]. However a subsequent interlaboratory trial found the sensitivity of this kit to be low [15]. In 2007 the WOAH certified another indirect iELISA (Platelia^TM^ Rabies II kit *ad usum Veterinarium*) for rabies serological testing (https://web.oie.int/VCDA/eng/Registre/Abstract%20sheet OIE%20 Register Platelia RabiesII v1.pdf). This kit was developed by Bio-Rad (Marnes-La-Coquette, France) and ANSES Nancy Laboratory, and was intended for use in serological testing of pets as part of travel schemes and for follow-up investigation in wildlife following oral vaccination [17]. The Platelia^TM^ Rabies II kit has also been validated for use in human samples [12], [18]. Previous studies conducted to evaluate the performance of this test have found excellent specificity over 99% [7], [12], [18], [19] but the sensitivity varied widely from 32% to 89% [7], [19], thus highlighting the need to re-evaluate its diagnostic capacity, especially under field conditions.

To our knowledge, this is the first study to evaluate, under field conditions, the performance of the Platelia^TM^ Rabies II kit *ad usum Veterinarium* (BioRad, *Inc*) for the detection and quantification of rabies virus antibodies. The study used serum samples collected from vaccinated and unvaccinated dogs in northern Tanzania. We compared the results of this kit relative to the standard reference FAVN test.

## 2 Materials and methods

### 2.1 Samples

Serum samples used in this study were drawn from a pool of samples collected in November 2017 in an immunogenicity study [20]. In brief, the immunogenicity study was carried out to investigate the potency of a rabies vaccine following storage within a locally made vaccine cooling storage device (Zeepot) with those stored in refrigeration units under cold chain conditions. In total, 412 domestic dogs that had not been previously vaccinated against rabies were enrolled in the study. Unvaccinated (day-0) blood samples, (n = 412) were collected from all dogs before they were randomly assigned to receive cold chain stored vaccine (n = 205) or Zeepot stored vaccine (n = 207). The study animals involved individuals of all age groups and of all body condition scores (BCS). The BCS was assessed and recorded according to the scale of 1 to 5 (1 - emaciated, 2 - underweight, 3 - ideal condition, 4 - overweight and 5 - Obese) as described by Baldwin and colleagues [21]. Vaccinated (day-28) blood samples (n = 345) were obtained from (n = 175) dogs that received cold chain stored vaccines and (n = 170) Zeepot stored vaccines. Following collection, the blood samples were kept in a cooler box for eight hours to separate the serum. Subsequently, the serum samples were collected and placed in sterile tubes labeled sequentially with a unique identification number (ID). The sample IDs, together with metadata detailing whether the samples were from animals that had been vaccinated or not, were also entered into a spreadsheet and finally the samples were stored at −20 °C for 105 days before testing.

### 2.2 Sample selection and preparations

Vaccinated (n = 101) samples were selected from the pool of day-28 samples obtained from dogs vaccinated with cold chain stored vaccine. The unvaccinated samples (n = 100) were drawn from the pool of day-0 samples. The list of test samples to be selected was produced using a random number generator. This was accomplished by entering the range of the sample IDs (min and max) in a random number generator and then specifying the number of observations to be generated. The randomly generated sample IDs were retrieved and recorded. The samples were subjected to heat treatment at 56 °C for 30 min. Each sample was then tested using both iELISA and FAVN.

### 2.3 iELISA test

The Platelia® Rabies II Kit *ad usum veterinarium* (iELISA) test was performed at the Nelson Mandela African Institution of Science and Technology in Tanzania. The iELISA kit and the test reagents were purchased from the Bio-Rad supplier (SMC® Ltd, UK, https://www.smcltd.com/). The procedure was carried out as previously described [17]. In brief, assays on the 201 samples were performed in 96 well microplates coated with rabies virus. All samples (including controls R4a, R4b, R3 and OIE serum) were diluted 1:100 and 100 μL was distributed according to the manufacture’s pre-established distribution plan. R4b (4 EU/ml) and OIE (4 IU/ml) were prepared with a two-fold serial dilution in order to obtain the concentration ranges 2, 1, 0.5, 0.25 and 0.125 EU/ml, and were dispensed as per microplate layout plan. Plates were incubated for 60 min at 37 °C, followed by washing with diluted wash solution (R2). The presence of immune complex was detected by the addition of 100 μL of protein A-Peroxidase and purified bovine protein conjugate (R7) into each well. Plates were then incubated for 60 min at 37 °C. A 100 μL of the diluted enzymatic development solution (R8 + R9) was distributed in each well and incubated in the dark for 30 min at room temperature. The colour reaction was stopped with the addition of 1 N H_2_SO_4_. Plates were read dichromatically at 450 and 595 nm. The estimation of the titre in EU/ml was calculated using the spreadsheet supplied with the kit. The conditions of validation for results were completed as described by the manufacturer. 1 EU/ml is ∼ 1 IU/ml, thus to compare with the results obtained by FAVN technique, a threshold protection of 0.5EU/ml was adopted to differentiate seropositive and seronegative dogs.

### 2.4 Standard reference fluorescent antibody virus neutralization test

Two hundred serum samples were sent to the Animal and Plant Health Agency in the UK for serological testing using the FAVN assay. The samples from Tanzania were shipped to the APHA laboratory, and the transportation took two days. Whilst being transported, the samples were packed with dry ice (UN 1845) to ensure preservation and integrity. The samples were placed in leak-proof cryo vials (primary containers) within a sealed biobag (secondary containers) with absorbent material between the two containers. The secondary containers were surrounded by dry ice packs and enclosed in a strong outer packaging. The FAVN procedure was carried out as previously described [22]. In brief, threefold serial dilutions of the positive and negative serum controls as well as of the test serum was prepared in the microplates. Each serum sample was added to four adjacent wells and serially diluted four times. A 50-µL of a dilution of challenge virus standard (CSV-11) containing 50–200 TCID50/ml was then added to each serum dilution well. The microplates were incubated for 1-hour at 37 °C in a humidified incubator with 5% CO_2_. Following incubation, 50-µL of the cell suspension with concentration of 4 x10^5^ BHK-21 cells/ml was added to each well and further incubated for 48-hours at 37 °C in a humidified incubator with 5% CO_2_. After incubation, the plates were fixed in 80% acetone, dried and stained with fluorescein isothiocyanate (FITC) anti-rabies monoclonal globulin to each well. For each serum dilution, four wells were examined for the presence or absence of virus-infected cells. The 50% endpoint of the antibody (D50) content of test sera and virus titrations (TCID50) were calculated according to the Spearman-Kärber method. By international convention, this titre was converted to a value in IU/mL by comparison of results obtained with those of the positive standard. The level of rabies neutralizing antibodies was expressed in International Unit per millilitre (IU/ml). A cut off value of 0.5 IU/ml was used to determine seroconversion as recommended by WHO [5].

### 2.5 Statistical analysis

The effectiveness of iELISA was evaluated using two tailed Spearman correlation analysis by comparing it with FAVN. The titres obtained by FAVN and iELISA (IU/mL AND EU/mL values, respectively) as well as the absorbance were log transformed to calculate the correlation coefficient. The influence of BCS on seroconversion was examined by fitting BCS as a predictor in binomial generalized linear mixed models (GLMM). The strength of association between BCS and seroconversion was estimated as odds ratios (ORs) with 95% confidence intervals. A ROC curve was plotted to verify the performance of the Platelia® Rabies II Kit over a range of possible cut-off values. All statistical analysis and regression fittings were performed using the statistical programming language, R version 3.5.3 [23].

## 3 Results

The iELISA and FAVN results of the 201 serum samples are shown in Table 1.

**Table 1:** Results of FAVN and iELISA.

		**FAVN (IU/ml)**						
		(a)				(b)		
		Unvaccinated				Vaccinated		
		≥ 0.5	< 0.5	n		≥ 0.5	< 0.5	**n**
**ELISA (EU/ml)**	≥ 0.5	1	1	**2**		81	3	**84**
	< 0.5	1	97	**98**		3	14	**17**
	**n**	**2**	**98**	**100**		**84**	**17**	**101**

**Fig. 1:**
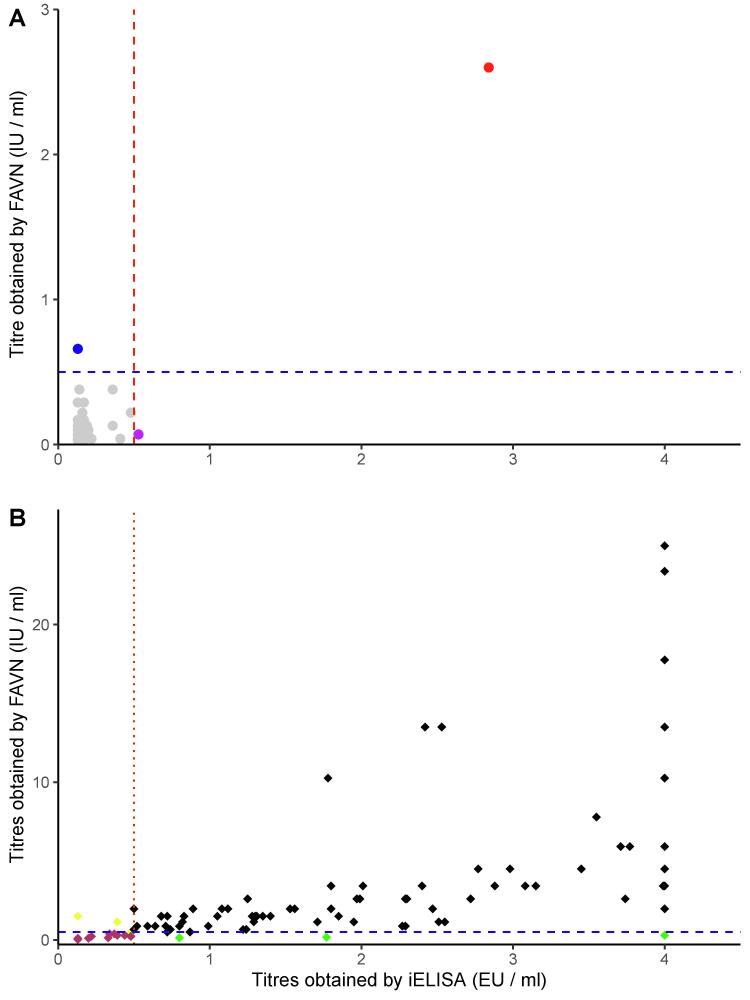
Antibody titres obtained with iELISA and the FAVN tests for vaccinated and unvaccinated dogs. The dashed blue line and dotted red line indicate the minimum seroconversion titres for each test. Panel A shows titres from unvaccinated dogs; grey dots show samples that tested negative for both tests, the blue dot is a sample that tested positive for FAVN test but negative for iELISA, the red dot is a sample that tested positive for both tests, whilst the purple dot is a sample that tested positive for iELISA and negative for FAVN. Panel B shows titres obtained from vaccinated dogs: black diamonds show samples that tested positive for both tests, maroon diamonds show samples that tested negative for both tests, yellow diamonds show samples that tested positive for FAVN test but negative for iELISA and green diamonds show samples that tested negative for FAVN test but positive for iELISA. (For interpretation of the references to colour in this figure legend, the reader is referred to the web version of this article.)

**Unvaccinated dogs:**Table 1a shows the immunoassay results for unvaccinated dogs. Following the FAVN assay, 98 out of 100 samples in the unvaccinated group were negative with titres less than 0.5 IU/ml (Fig. 1A, grey & red dots). Of these 98-gold standard negative samples, the iELISA assay correctly identified 97 as unvaccinated giving a specificity of 99.0%, and incorrectly identified one sample (Fig. 1A, purple dot) as vaccinated with an iELISA titre just above the cut-off line (0.53 EU/ml). Of the two unvaccinated samples that had FAVN titres ≥ 0.5 IU/ml (Fig. 1A, blue & red dot), one was identified as unvaccinated with the iELISA (Fig. 1A, blue dot), whilst a second was identified as vaccinated with both the iELISA and the FAVN test (Fig. 1A, red dot).

**Vaccinated animals:**Table 1(B) and Table 2 shows the immunoassay results for vaccinated dogs. Interestingly, among the 101 vaccinated samples, both the iELISA and FAVN assays determined 17 samples with titres below 0.5 IU/ml (Table 2). However, upon further analysis in Table 1B, it was revealed that out of these 17 samples, only 14 were consistent between both tests in having titres below 0.5 IU/ml (maroon diamonds). . Three samples had titres < 0.5 IU/ml for FAVN test but ≥ 0.5 EU/ml for iELISA (green diamonds), three samples had titres ≥ 0.5 IU/ml for FAVN test but < 0.5 EU/ml for iELISA (yellow diamonds) and 81 had titres ≥ 0.5 IU/ml for both tests (black diamonds) (Fig. 1B). Following the iELISA assay, 81 out of the 83 gold-standard positive samples were recorded as positive giving a sensitivity of 98.0%.

**Table 2:** The level of rabies antibodies detected by iELISA and FAVN tests in vaccinated dog sera.

		**Elisa**	**FAVN**
**Category** **(Seroconversion)**	**Antibody level (EU/ml or IU/ml)**	**n**	**n**
Undetectable	< 0.125	5	5
Insufficient	0.125 to < 0.5	12	12
Sufficient	0.5 to 4	63	59
High	> 4	21	25
Total		101	101

### 3.1 Receiver operating characteristic (ROC) analysis

**Fig. 2:**
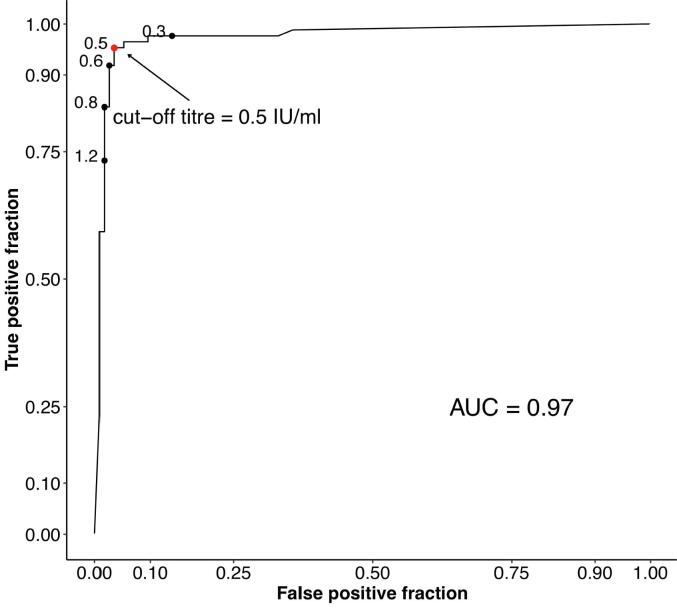
ROC curve showing the optimal (0.5 IU/ml) cut-off value for the Platelia^TM^ Rabies II test. (For interpretation of the references to colour in this figure legend, the reader is referred to the web version of this article.)

The ROC curve is shown in Fig. 2. As can be seen from the annotations, the optimal cut-off value for the iELISA assay was 0.5 IU/ml. The ROC curve shows an AUC of 0.97 (95% confidence interval (CI) 0.95–1.00), demonstrating that the Platelia Rabies II test accurately discriminates between vaccinated and unvaccinated dogs.

**Fig. 3:**
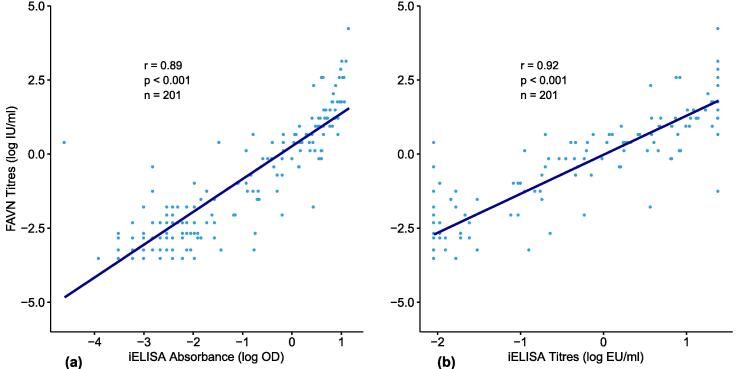
Spearman correlation analysis of the FAVN and iELISA tests performed on the dog sera.

Spearman correlation between FAVN and iELISA are shown in Fig. 3. A strong correlation (r = 0.89) was observed when iELISA was expressed as absorbance (O.D). (Fig. 3a). The correlation increased (r = 0.92) when iELISA titres were expressed as EU/ml (Fig. 3b). The linearity was verified by the regression lines, and in both scenarios the correlation between the two tests was statistically significant (p < 0.001).

### 3.2 Assessment of the impact of body condition score on immunogenicity of vaccinated dogs

The FAVN results and the associated BCS of the dogs from which the samples were collected has been summarized in Table 3. No dogs with BCS 4 or 5 were recorded in the study.

**Table 3:** Distribution of BCS and the seroconversion rate in the vaccinated group as analyzed by FAVN.

BCS	N	Seroconverted	%Seroconversion
1	15	9	60
2	67	58	87
3	19	17	90

The majority (67%) of dogs had BCS of 2. As BCS increased, so did the percentage of dogs that seroconverted (Table 3, Table 4). The odds of seroconversion of dogs with BCS of 2 were 7.00 times higher than that of dogs with BCS of 1 (OR: 7.00; 95% CI: 1.87 – 26.27). The difference was statistically significant p < 0.05. Similarly, dogs with BCS of 3 had 7.00 times higher odds of seroconversions than dogs in a baseline group (OR: 7.00; 95% CI: 1.10 – 44.60).

**Table 4:** The results of GLMM showing the relationship between BCS and seroconversion.

**Variable**	**Odd ratio**	**95% CI**	**P value**
BCS 2	7.00	1.87–26.27	0.004
BCS 3	7.00	1.10–44.61	0.04

**Fig. 4:**
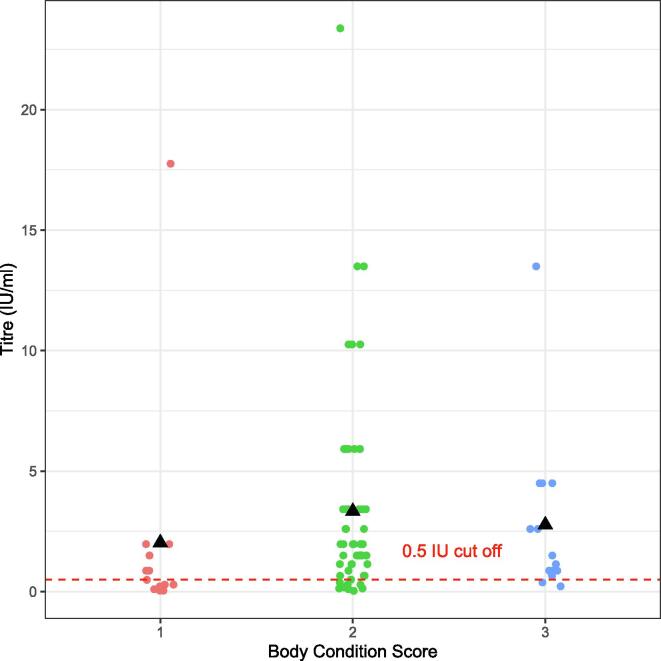
Dot-plot showing the BCS and antibody level titres (IU/ml) of dogs in the vaccinated group. The dashed line indicates the minimum seroconversion titre. Black triangle shapes show the mean titres for each BCS.

Quantitatively, dogs with BCS of 2 and 3 elicited higher antibody titres than those with BCS of 1 (Table 4 & Fig. 4).

## 4 Discussion

This paper presents the performance of the iELISA (Platelia^TM^ Rabies II Kit, Bio-Rad) for detection and titration of anti-rabies antibodies in comparison to the conventional reference method of seroneutralisation (FAVN) on samples collected from vaccinated and unvaccinated domestic dogs living in Tanzanian villages. An iELISA cut-off value of 0.5 EU/ml, which is harmonised with 0.5 IU/ml threshold [24], was adopted to simplify interpretation of results with those obtained using seroneutralisation assays. The findings from our study indicate an excellent overall performance of the iELISA when compared to the FAVN (Fig. 2, Fig. 3).

Our data demonstrate a strong correlation between titres measured by iELISA and the FAVN test (r = 0.92, p < 0.001). The ROC curve indicated that the performance of the iELISA was excellent (AUC 0.97, 95% CI 0.95–1.00) (see Fig. 2) and was able to discriminate between vaccinated and unvaccinated individuals with a high degree of accuracy. The iELISA was found to have a diagnostic specificity of 99%, in agreement with studies carried out previously [7], [18], [19], [25] in which the specificity of the iELISA was reported to be between 99.4 and 100%. These results shows that the Platelia^TM^ Rabies II Kit iELISA test can accurately determine unvaccinated dogs.

Besides having excellent specificity, the results obtained from our study demonstrated that the iELISA has high sensitivity of 98%. This result is similar to that reported by Feyssaguet *et al* (98.6%) who evaluated the perfomance of the same iELISA on human samples [12], and higher than that described in domestic carnivores by Servat *et al* (83%) [17] and Wasniewski *et al*. (78.2%) [7]. Interestingly, our results differed from those of Knoop *et al*. who reported the iELISA sensitivity to be just 32.4% when analysing wild carnivore sera. However in the same work of Knoop and colleagues, the iELISA showed improved sensitivity of 83.9% when samples from laboratory animals were analysed [19]. The authors suggested that discrepancies and unsatisfactory results of the iELISA could have been caused by i) lack of standardized formula needed to convert OD to EU/ml, ii) poor quality of samples and frequent freeze–thaw cycles that may have led to the degradation of antibodies, subsequently diminishing the test sensitivity [26], [27], and iii) high purification of the glycoprotein G (extraction from the membrane can damage the immunogenic glycoprotein G leading to loss of sensitivity) [19], [7].

Previous studies which have evaluated the performance of the Platelia® Rabies II Kit in domestic carnivores used serum samples submitted to the laboratory for serological testing either in the context of international trade or international animal movement [7], [17], [19], [25], [28]. In addition, these studies used a relative large sample size (n > 1000) [12], [17]. In the current study, the evaluation of the iELISA was carried out on serum samples collected on dogs (n = 201) in field settings in East Africa. Despite the difference in sample size and in the originality of samples, our results are similar to those from the studies above suggesting that the performance of the iELISA is robust across a range of settings.

Our data show BCS had a positive influence on the likelihood of seroconversion (Table 3, Table 4). This finding aligns with our previous work [20] in which we demonstrated a positive linear trend between BCS and seroconversion suggesting dogs of poor condition may not respond as expected to vaccination. Notably, throughout the study period, we did not observe any overweight (BCS 4) or obese (BCS 5) dogs. Instead, a significant proportion of dogs (67%) were classified as underweight (BCS 2). These findings were also reported by Czupryna *et al* who, in a study also in northern Tanzania, did not identify any overweight or obese dogs and found that 70% of dogs were underweight [29]. Comparably poor body condition of dogs has been reported in other studies conducted in rural areas of Africa such as Chad [30], Ethiopia [31], Uganda [32], and Zambia [33]. These results may be explained by the fact that dogs in these countries often lack access to proper veterinary care and adequate nutrition compared to their counterparts in ‘global north’ countries. In rabies-endemic regions, dogs are typically not fed commercial food; instead, they frequently scavenge for food, for example in rubbish pits [34], [35], [36]. Additionally, dogs in rural areas often have little or no access to veterinary services [32], [33] which results in a higher prevalence of infectious diseases, including polyparasitism [37], [38]. Polyparasitism negatively impacts dog health [2], [39], [40] and can significantly affect the immune response following vaccination [41]. In their study, Bahloul *et al* inferred poly-parasitism and malnutrition as a possible cause for insufficient immune response [42].

Surprisingly, we found 14 dogs in the vaccinated group had titres below the threshold level in both FAVN and iELISA tests (Table 1b & Fig. 1B, maroon diamonds). Half of these dogs (50%) had a BCS of 1, while 43% had a BCS of 2. A study conducted in Indonesia [43] found that BCS was associated with the loss of adequate levels of binding antibodies, with approximately 50% of dogs with a BCS lower than three experiencing a decline in binding antibodies below the adequate level by day 90 post vaccination. This finding led to their conclusion that BCS was an important factor in determining the duration of immune response [43]. We therefore hypothesise that poor body condition could be a reason for these vaccinated dogs having low titres (Table 3 and Fig. 4), however we cannot rule out other factors such as current infections contributing to this outcome.

A limitation of our study, inherent to dog populations in much of sub-Saharan Africa, is that the majority of dogs have the same BCS (equal to 2). Consequently, when investigating the relationship between BCS and antibody response levels, dogs with BCS 1 and 3 become more influential due to their relative rarity and can affect results depending on the cutoff selected for classifying individuals as unvaccinated and the removal of outliers. For example, one dog with BCS of 3 had a baseline titer (day-0) of 0.38 IU/ml, close to the widely accepted threshold of 0.5 IU/ml for vaccination status, and its high post-vaccination titer (day-28) of 70.13 IU/ml, inflated the group's mean. Due to its disproportionate influence, we classified this individual as previously exposed (vaccinated) and thus excluded it from the analysis. Studies with a more diverse range of dog BCS values would not encounter this challenge, and increasing the sample size may prove helpful in future research on this question.

There were three samples from vaccinated dogs that had FAVN titres < 0.5 IU/ml but had iELISA titres ≥ 0.5 EU/ml (Fig. 1B, green diamonds). All three of these samples had FAVN titres close to the threshold value. The possible explanation for this discordance can be linked to what is measured by each assay. The BioRad iELISA measures IgG that binds to the rabies glycoprotein rather than viral neutralization. High binding antibody will be detected, however not all antibodies that bind to G in its native form neutralize virus, thus the titres obtained from ELISAs can be slightly higher than those of the FAVN. In this study all blood sampling was performed at four weeks post vaccination when IgG is expected to be the primary immunoglobulin and is thus a time point well suited for ELISA measurements.

The FAVN detected two individuals in the unvaccinated group having titres ≥ 0.5 IU/ml. There are a number of possible explanations for this: i) the information provided by the owner that the dogs were not vaccinated might have been wrong; ii) potential maternal transfer of antibody to puppies [44] and iii) the dogs had been previously exposed to inactivated rabies virus, perhaps through scavenging carcasses of animal that had died of rabies [45]. Indeed, several studies have shown approximately 5% of unvaccinated dogs in rabies endemic countries such as Tanzania to be sero-positive to rabies virus [46], [47].

## 5 Conclusions

In conclusion, the findings from our study indicate that the results obtained by iELISA are comparable to the FAVN test and, given the use of the G protein in the iELISA assay, are likely to be correlated with protection. Our data have demonstrated that the iELISA is similarly sensitive and specific as the FAVN test. Given the iELISA is much easier to administer, these results suggest that the iELISA could become a valuable and reliable tool to check vaccination status for screening animals in vaccination programs.

## Author Contributions

**Ahmed Lugelo:** Conceptualization, Data curation, Formal analysis, Investigation, Methodology, Project administration, Supervision, Validation, Visualization, Writing – original draft, Writing – review & editing. **Katie Hampson:** Funding acquisition, Project administration, Supervision, Validation, Writing – review & editing. **Lorraine M McElhinney:** Methodology, Validation, Writing – review & editing. **Felix Lankester:** Conceptualization, Formal analysis, Funding acquisition, Investigation, Methodology, Project administration, Supervision, Validation, Visualization.

## Funding

This study was funded by the Department of Health and Human Services of the National Institutes of Health [R01AI141712] and MSD Animal Health. The content is solely the responsibility of the authors and does not necessarily represent the official views of the National Institutes of Health. KH is funded by Wellcome [207569/Z/17/Z]. LM is funded by Defra, the Scottish Government and Welsh Government, through grants SV3500 and SE0433.

## Declaration of Competing Interest

The authors declare that they have no known competing financial interests or personal relationships that could have appeared to influence the work reported in this paper.

## Data Availability

Data will be made available on request.
